# Justification of empiric methodology to determine dexmedetomidine dose for the TREX study

**DOI:** 10.1111/pan.14605

**Published:** 2022-12-04

**Authors:** Nicola Disma, Bianca M. Goffredo, Sara Cairoli, Ginevra Cirillo, James Morse, Brian J. Anderson, the TREX (Trial Remifentanil DEXmedetomidine) Italian Investigators, Rachele Bonfiglio, Ramona Cordani, Marco Garrone, Elisa Patrone, Annalisa Iengo, Paola Bocca, Francesca Izzo, Veronica Diotto, Elena Lenares, Chiara Robino, Simona Neri, Laura Colantonio, Edoardo Calderini, Sergio Picardo, Isabella Tucci, Alessandra Di Persio, Luigi Montagnini, Luca Blesi, Barbara Pistone, Beate Kuppers, Brita De Lorenzo, Fabio Caramelli, Lorena Pasini

**Affiliations:** ^1^ Unit for Research and Innovation, Department of Anaesthesia IRCCS Istituto Giannina Gaslini Genova Italy; ^2^ Division of Metabolic Disease and Drug Biology IRCCS Ospedale Bambino Gesù Rome Italy; ^3^ Department Anesthesiology, Faculty Medicine and Health Science University of Auckland Auckland New Zealand

**Keywords:** dexmedetomidine, neuronal apoptosis, neuroprotection, pharmacodynamics, pharmacokinetics

## Abstract

**Introduction:**

Dexmedetomidine is the sedative agent administered in combination with remifentanil and low dose of sevoflurane in the interventional arm of the ongoing TREX trial (**T**rial **R**emifentanil **DE**xmedetomidine). The TREX pilot study (published in Paediatr Anaesth 2019;29:59–67) established infusion rates higher than those initially proposed. This could be attributed to an inappropriate target concentration for sedation or incorrect initial pharmacokinetic parameter estimates.

**Methods:**

The TREX study is a Phase III, randomized, active controlled, parallel group, blinded evaluator, multicenter, superiority trial comparing neurological outcome after standard sevoflurane anesthesia with dexmedetomidine/remifentanil, and low dose sevoflurane anesthesia in children aged less than 2 years undergoing anesthesia of 2 h or longer. In this report, dexmedetomidine pharmacokinetics were analyzed in the interventional arm of the Italian population.

**Results:**

There were 162 blood samples from 32 infants (22 male and 10 female). The median (IQR) age was 12 (5.2–15.5) months, weight 9.9 (7.3–10.8) kg. Duration of anesthesia ranged from 2 to 6 h. None of the children were born premature (median postnatal age 39 weeks, IQR 38–40 weeks). A 3‐compartment PK model that incorporated allometric scaling and a maturation function demonstrated plasma concentration observations from the current Italian arm of the TREX study were consistent with those predicted by a “universal” model using pooled data obtained from neonates to adults.

**Conclusions:**

This current PK analysis from the Italian arm of the TREX study confirms that plasma concentration of dexmedetomidine is predictable using known covariates such as age and size. The initial target concentration (0.6 μg.L^−1^) used to sedate children cared for in the intensive care after cardiac surgery was inadequate for infants in the current TREX study. A target concentration 1 mcg.L^−1^, corresponding to a loading dose of 1 mcg.kg^−1^ followed by an infusion of 1 mcg.kg^−1^.h^−1^, provided adequate sedation.

**What is already known about this subject**

- Dexmedetomidine has neuroprotective properties and is used for sedation in children enrolled in the TREX study
- The TREX pilot study identified that higher dexmedetomidine infusion rates were required than those initially proposed. This could be attributed to an inappropriate target concentration for sedation or incorrect initial pharmacokinetic parameter estimates.

**What this study adds**

- The pharmacokinetic parameter estimates from the Italian arm of the TREX study are similar to initial estimates used in the pilot study. Pooling these observations with published data from neonates to adults strengthened a “universal” dexmedetomidine pharmacokinetic model.
- The initial dexmedetomidine target concentration (0.6 μg.L^−1^) used in the TREX pilot study was inadequate for sedation. Higher doses (1.0 μg.kg^−1^ loading dose, followed by an infusion of 1.0 μg.kg^−1^.h^−1^), reflecting a higher target concentration of 1 μg.L^−1^, proved adequate.

## 1 INTRODUCTION

TREX (trial of remifentanil and dexmedetomidine) is an ongoing randomized controlled study to assess whether an anesthetic regime including regional blockade with dexmedetomidine‐remifentanil sedation (and low inspired sevoflurane concentration) has equal or superior long‐term neurological effects compared to children undergoing anesthesia for two hours or longer given sevoflurane alone with regional blockade (ClinicalTrials.gov Identifier: NCT03089905; EudraCT number: 2017‐002803‐81). The study follows examination of cognitive impairment in infants after short duration anesthesia (approximately 1 h) that showed no evidence of clinical anesthetic toxicity.^1^, ^2^, ^3^, ^4^ Dexmedetomidine was chosen for sedation because preclinical evidence suggests neuroprotective effects.^5^ Current TREX drug protocols were established after a pilot study to ascertain feasibility and safety of dexmedetomidine and remifentanil based anesthesia.^6^

A secondary outcome of the TREX study is to examine pharmacokinetics (PK) of dexmedetomidine and its complex pharmacodynamic (PD) interactions with remifentanil and sevoflurane in children. Permission for the Italian arm of the study was conditional on review of dexmedetomidine pharmacokinetics. We describe dexmedetomidine PK in children allocated to the interventional arm of the current TREX study and discuss protocol changes implemented upon completion of the pilot study relating to the target concentration for adequate sedation.^6^

## 2 METHODS

The TREX study is a Phase III, randomized, active controlled, parallel group, blinded evaluator, multicenter, superiority trial comparing standard sevoflurane anesthesia with dexmedetomidine/remifentanil and low‐dose sevoflurane anesthesia in children aged under 2 years undergoing anesthesia of at 2 h or longer. Dexmedetomidine 1 μg.kg^−1^ and remifentanil 0.5–1 μg.kg^−1^ were given over 10 min. Maintenance of anesthesia was dexmedetomidine 1 μg.kg^−1^.h^−1^, remifentanil 0.1–0.8 μg.kg^−1^.min^−1^, and end‐tidal sevoflurane 0.8 vol%. Blood samples were taken from an indwelling cannula following dexmedetomidine administration at five time intervals (T0: baseline; T1: 10–15 min after loading dose; T2: 30 min after loading dose; T3: mid surgery; T4: end of surgery; T5: 20 min after infusion end of dexmedetomidine infusion) and collected into a lithium heparin tubes. In any event of suspected light anesthesia in the interventional arm (defined as purposeful movements and/or hypertension) rescue administration of remifentanil, dexmedetomidine, increased sevoflurane dose, neuromuscular blockade, or a propofol bolus were allowed at discretion of the anesthetist in charge and recorded in the case report form.

### 2.1 Dexmedetomidine assay

The coordinating center for the current Italian TREX is IRCCS Istituto Giannina Gaslini. Eight centres are participating this TREX in Italy. Plasma was separated by centrifugation (2500 *g* for 10 min at 4°C) and stored at −80°C at the site until assay. The assay was performed by an accredited central laboratory, located at IRCCS Ospedale Pediatrico Bambino Gesù in Rome (Italy). Liquid chromatography and mass spectrometry analysis were performed by a UHPLC Agilent 1290 Infinity II 6470 (Agilent Technologies) equipped with an ESI‐JET‐STREAM source operating in the positive ion (ESI+) mode for Dexmedetomidine. The software used for controlling this equipment and analyzing data was MassHunter Workstation (Agilent Technologies).

Bias (%) and precision (% coefficient of variation, CV) for High, Medium, and Low ranges were: Bias −1.02%, 4.14% and 6.08%; CV was 2.05%, 1.93%, 4.19%. Lower limit of detection was 0.15 μg.L^−1^.

### 2.2 Pharmacokinetic modeling

Italian dexmedetomidine concentration data were pooled with published dexmedetomidine time‐concentration observations: Potts,^7^, ^8^ Cortinez,^9^ Rolle,^10^ Talke.^11^ Analysis of these pooled data without the additional observations from Italian children, are available and details of data sources, pharmacokinetic analysis, and results are published.^12^ The NMTRAN (NonMem TRANslator—converts the data file and the stream into FORTRAN code file for use by NMTRAN) is available in Appendix S1. A three‐compartment PK models with first order elimination were used to describe dexmedetomidine pharmacokinetics. The model was parameterized in terms of clearance (CL), volumes of distribution (V1, V2, V3), and intercompartmental clearances (Q2, Q3). Allometric theory was used to quantify size‐related changes in PK parameters. PK parameters (e.g., CL, Q2, Q3, V1, V2, V3) were standardized to an adult measure of body size (Fsize) with a standard weight of 70 kg using allometric scaling (Equation 1).^13^, ^14^, ^15^

(1) $$\text{Fsize}={\left(\frac{\text{size}}{70}\right)}^{\mathrm{EXP}}$$

where *Fsize* is a variable describing the fractional difference from a standard adult value and EXP is the allometric exponent; ¾ for functional processes such as clearance and 1 for volumes.

The maturation of dexmedetomidine clearance was assessed using a maturation function (MF, Equation 2)

(2) $$\mathrm{MF}=\frac{{\mathrm{PMA}}^{\text{Hill}}}{{\mathrm{TM}}_{50}^{\text{Hill}}+{\mathrm{PMA}}^{\text{Hill}}\;}$$

where PMA is the postmenstrual age in weeks; TM_50_ is the maturation half‐time, and the Hill exponent relates to the steepness of the maturation profile.^16^

Equation 3 shows how Fsize can be used to scale a standard value of CL (CL_STD_) and account for maturation with age to predict the group CL.

(3) $$\mathrm{CL}={\mathrm{CL}}_{\mathrm{STD}}\times \;\text{Fsize}\times \mathrm{MF}$$

Population parameter variability (PPV) was accounted for using an exponential model for the random effect variables (η). This assumes a log‐normal distribution and avoids parameter estimates falling below biologically plausible values. Variables were assumed to have a mean of zero and variance denoted by ω^2^ (Equation 4).

(4) $$P_{i}=P_{\mathrm{TV}}e^{\mathrm{\eta i}}$$

where P is the parameter (e.g., CL) for the *i*th individual, P_TV_ is the typical value for that parameter and η is the random effects variable.

Residual unidentified variability (RUV) was modeled using both proportional and additive residual errors (Equation 5). The between subject variability (η_RUV,i_) of the RUV was also estimated for data.

(5) $$\mathrm{SD}\mathrm{ij}=\sqrt{\left({\left({\mathrm{Obs}}_{\mathrm{ij}}.\theta_{\mathrm{RUV}\_\mathrm{CV}}\right)}^{2}+{\left(\theta_{\mathrm{RUV}\_\mathrm{SD}}\right)}^{2}\right).}e^{\eta {\mathrm{PPV}}_{\mathrm{RUV}\;i}}$$

where Obs_*ij*_ is the dexmedetomidine plasma concentration in the *i*th individual at the *j*th time. Individual predictions of dexmedetomidine concentration were calculated using Equation 6 with the random effects (ε) fixed to 1.

(6) $$Y={\mathrm{Obs}}_{\mathrm{ij}}+\mathrm{SD}\mathrm{ij}.\varepsilon$$

Population parameter estimates were obtained using nonlinear mixed effects models (NONMEM 7.5 ICON Development Solutions, USA) with first‐order conditional estimation and a convergence criterion set to three significant digits. The population mean parameters, between subject variance and residual variance were estimated using the first order conditional interaction estimate method using ADVAN13 TOL = 9 of NONMEM.

### 2.3 Model selection

The minimum value of the objective function (OBJ [−2log‐likelihood (−2LL)]) provided by NONMEM served as a guide during model building. Model selection was also based on parameter plausibility and prediction‐corrected visual predictive checks (PC‐VPC) plots,^17^ where observations and simulations are multiplied by the population baseline value divided by the individual‐estimated baseline. For two nested models, a decrease in the minimum value of the objective function (ΔOBJ) of 3.84 points for an added parameter was considered significant at the 0.05 level. Shrinkage considers the quality of the observed data. The term η‐shrinkage refers to the between subject variability. When the observed data are informative, η‐shrinkage approaches zero and when the data are less informative it approaches 1. Bootstrap methods provided a means to evaluate parameter uncertainty.^18^ A total of 1000 bootstrap replications were used to estimate parameter medians and confidence intervals. The results from the population models are presented as parameter estimates, together with their 95% confidence intervals (95% CI). Between subject parameter variability is expressed as an apparent coefficient of variation obtained from the square root of the variance estimate [CV (%)].

## 3 RESULTS

**FIGURE 1:**
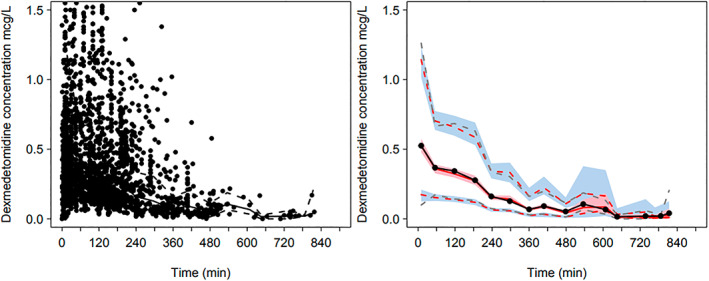
Prediction‐corrected visual predictive check (PC‐VPC) for the pooled dexmedetomidine pharmacokinetic model. Model developed using pooled pediatric^7^ and adult^11^, ^29^ dexmedetomidine plasma concentrations. Plots show median (solid) and 90% intervals (dashed lines). The left‐hand plot shows all prediction corrected observed dexmedetomidine concentrations. Right‐hand plot shows prediction corrected percentiles (10%, 50%, and 90%) for observations (gray dashed lines) and predictions (red dashed lines) with 95% confidence intervals for prediction percentiles (median, pink shading; 5th and 95th blue shading).

**FIGURE 2:**
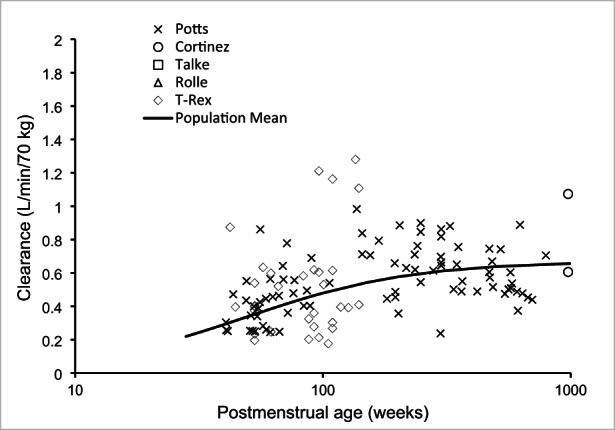
Maturation of dexmedetomidine clearance using pooled data. Individual post‐bayesian clearance estimates for individual studies are shown as discrete symbols. The solid line is the population mean.

Parameter estimates for the pooled data determining the dexmedetomidine model are shown in Table 1. There were 2267 dexmedetomidine concentrations that were amenable for modeling in the pooled dexmedetomidine PK analysis. A 3‐compartment PK model proved superior to the 2‐compartment model for the pooled dexmedetomidine analysis (ΔOBJ 285.2). The final model including allometric scaling pharmacokinetic parameters using total body weight. The increase in CL due to age accounted for with a maturation function. A pcVPC for the final model using pooled data is shown in Figure 1. Maturation of dexmedetomidine clearance scaled using total body weight is shown in Figure 2.

**TABLE 1:** Dexmedetomidine population pharmacokinetic parameter estimates

	Estimate	PPV (%)	Bootstrap median	95% CI	Sh%
V1 (L/70 kg)	19	105	18.7	15.2, 24.1	25.6
V2 (L/70 kg)	24.1	35.2	25.2	19.9, 31.3	27.4
V3 (L/70 kg)	50	63.4	50.6	42.8, 66.8	2.0
CL (L/min/70 kg)	0.67	37.7	0.67	0.61, 0.75	1.0
Q2 (L/min/70 kg)	1.37	54.3	1.31	0.91, 1.70	14.3
Q3 (L/min/70 kg)	0.518	94.4	0.491	0.33, 0.71	3.3
TM_50_	48.9	‐	48.6	40.3, 66.8	‐
Hill	1.29	‐	1.29	0.76, 2.2	‐
Additive residual Error (μg/ml)	0.006	η_RUV_ 53	0.006	‐	‐
Proportional Residual Error (%)	20.7	‐	20.4	18.2, 22.6	‐

Abbreviations: CL; clearance; Hill: exponent describing the steepness of the maturation profile; PPV%; population parameter variability; Q; intercompartmental clearance; Residual unidentified variability: RUV; Sh% = shrinkage; TM_50_; maturation halftime; V: Volume of distribution.

*Note*: Size is accounted for using theory‐based allometric scaling to a 70 kg individual with the allometric exponents of ¾ for CL and 1 for V. PPV% = √variance. Bootstrap median and 95% confidence interval (CI) determined from 100 bootstrap replications.

**FIGURE 3:**
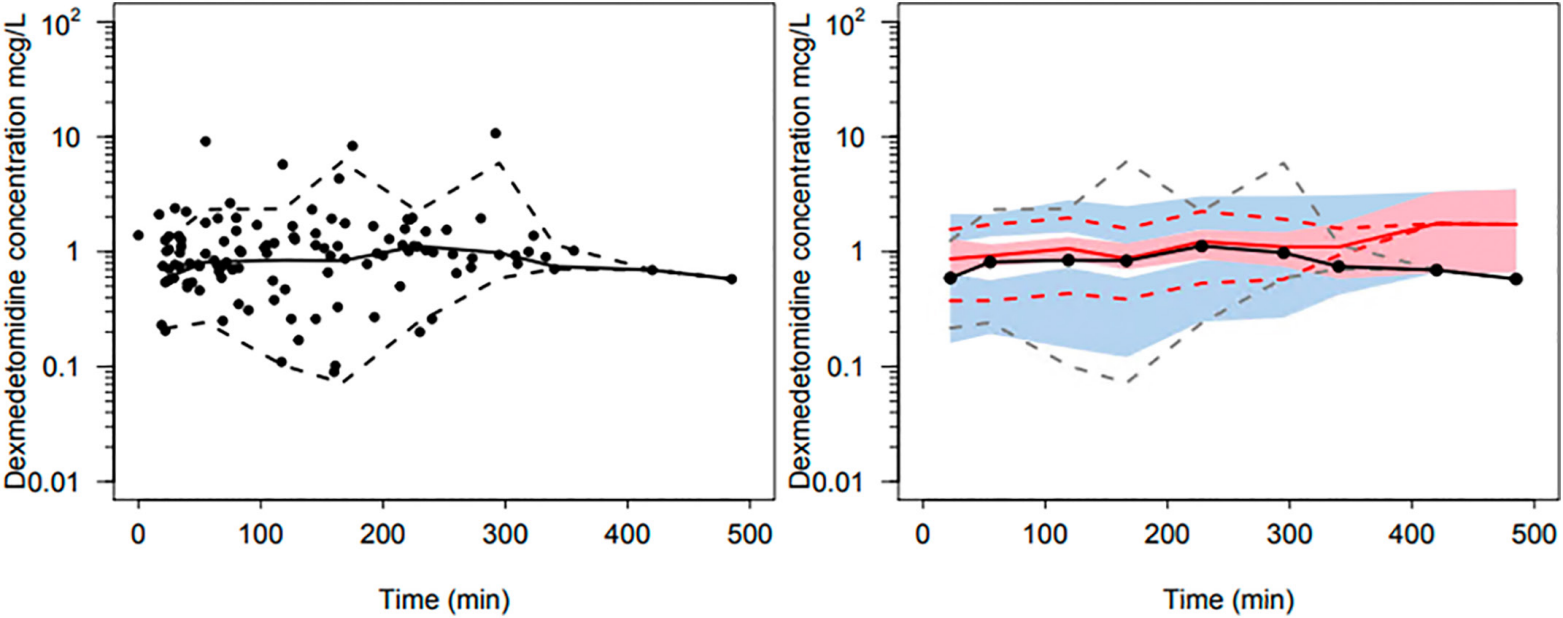
Prediction‐corrected visual predictive check (PC‐VPC) for the dexmedetomidine data obtained only in the TREX study using the pooled pharmacokinetic model. Plots show median (solid) and 90% intervals (dashed lines). The left‐hand plot shows all prediction corrected observed dexmedetomidine concentrations. Right hand plot shows prediction corrected percentiles (10%, 50%, and 90%) for observations (gray dashed lines) and predictions (red dashed lines) with 95% confidence intervals for prediction percentiles (median, pink shading; 5th and 95th blue shading).

This pooled data analysis included 120 blood samples from 32 patients (22 male and 10 female) from the Italian arm of the ongoing TREX study. The median (IQR) age was 12 (5.2–15.5) months, weight 9.9 (7.3–10.8) kg. None of children were born premature (median postnatal age 39 weeks, IQR 38–40 weeks). Children underwent anesthesia for at least 2 h, with the longest lasting 6 h. Dexmedetomidine measurements in blood samples range from undetectable concentrations up to 10.7 μg.L^−1^; there are 9 samples in six patients where the dexmedetomidine concentration was undetectable. Figure 3 demonstrates the time‐concentration predictions based on the pooled data model with the data from Italian children added as individual points. This figure shows that dexmedetomidine concentration observations from these children in the Italian arm of the TREX study were consistent with those predicted by the “universal” model using pooled data.

There were 11 events of light anesthesia that occurred in eight patients, with movements or hypertension observed during the surgical procedure. A plasma concentration was available in five of these infants and the mean plasma concentration before events of “light anesthesia” was 0.79 SD 0.18 μg.L^−1^. These events were resolved by increasing dexmedetomidine on two occasions, remifentanil on seven occasions, and sevoflurane on three occasions. Two drugs were increased simultaneously on three occasions. Propofol or rocuronium were used only once each.

## 4 DISCUSSION

The goal of pharmacologic treatment is the target effect. A pharmacodynamic (PD) model is used to predict the target concentration known to be associated with a target effect. Pharmacokinetic information is used to predict dose that achieves the target effect. That pharmacokinetic information should include covariates such body size and age (describing clearance maturation) as these are important to predict concentration in an individual child.^19^

The dosing schedule for the pilot TREX study^6^ was based on known pharmacokinetics in children with a mean age of 3.8 years (median 3 years, range 1 week‐14 years) and weight of 16.0 kg (median 13.3 kg, range 3.1–58.9 kg).^8^ It remained uncertain if parameter estimates in this cohort were similar to those in the current TREX cohort.

Dexmedetomidine dose infusion for the pilot TREX study were set to establish a steady‐state concentration of 0.6 μg.L^−1^. This target concentration was satisfactory for children nursed in an intensive care unit after cardiac surgery^8^ and has subsequently proven useful for sedation during radiological procedures where infants are children are subjected to minimal external stimuli.^20^ Higher infusion rates were entertained during the pilot study design but concern was expressed that cardiovascular adverse effects could cause compromise in children.^21^, ^22^, ^23^ There were few adverse effect concentration‐response relationships in children published at the time of trial initiation,^24^ although subsequent data have improved understanding of these adverse effects.^25^

A change in trial protocol to increase dexmedetomidine infusion rates was necessary during the pilot study because sedation was inadequate. Dose increased from a loading dose of 0.6 μg.kg^−1^ over 10 min with maintenance 0.6 μg.kg^−1^.h^−1^ (Version 1) to 1 μg.kg^−1^ over 10 min with maintenance 1 μg.kg^−1^.h^−1^ (Version 2) to 1 μg/kg over 10 min with maintenance 1–1.5 μg.kg^−1^.h^−1^ (Version 3).^6^ Hypotension increased with dose, but was generally mild and no child required vasoconstrictor rescue during the pilot study. Only one child suffered bradycardia and that episode was considered unrelated to dexmedetomidine.^6^ Consequently, the current TREX protocol suggests dexmedetomidine loading dose 1 μg.kg^−1^ followed by an infusion of dexmedetomidine at 1 μg.kg^−1^.h^−1^.

It remained uncertain if the increased dose required for sedation was due to pharmacokinetic differences in the cohort used for dose prediction^8^ or if a higher target concentration was necessary for adequate sedation. This current study confirms that pharmacokinetics in Italian children involved in the ongoing TREX study are similar to those of others^12^ including those children recovering after cardiac surgery. Although few pharmacodynamic studies in children have been performed, those in adults confirm a target concentration for sedation of 1 μg.L^−1^,^26^, ^27^ consistent with the higher doses required in the later protocols of the pilot study.^6^ Concentration at steady‐state approximated 1 μg.L^−1^, albeit with considerable variability (Figure 2). The cardiovascular compromise associated with rapid infusion have been demonstrated using simulation^28^ and cardiovascular changes observed in those children using pilot study protocol versions 2 and 3 could also have been predicted had concentration‐adverse effects been described before trial initiation.

The incidence of episodes of light anesthesia requiring a rescue dose of anesthetics is lower in our series of patients (11 episodes in 35 infants) than that of the pilot study (42 episodes in 60 patients). Dexmedetomidine plasma concentration when events of light anesthesia occurred was lower (0.79 μg.L^−1^) than the target concentration (1 μg.L^−1^), which could contribute to the occurrence of these events. There are other contributors to inadequate sedation, for example, inadequate caudal blockade, inappropriate upper limb movement. The current protocol also includes the use of low dose of sevoflurane, that was not used in the pilot study. It will be important to have a better understanding of the complex interaction between dexmedetomidine, remifentanil and sevoflurane, with the subsequent pharmacodynamic. This is one of the follow‐up steps of this current trial.

This current PK analysis from the Italian arm of the TREX study confirms that plasma concentration of dexmedetomidine is predictable using known covariates such as age and size. The target concentration sought in infants appears similar to that required for sedation in the adult population.^26^, ^27^

### APPROVAL STATEMENT

The TREX Study Steering Committee (*TREX Study Steering Committee*: Brian Anderson, Auckland Children's Hospital; Dean B. Andropoulos, Texas Children's Hospital; Ansgar Brambrink, Columbia University; Andrew Davidson and Katherine Lee, The Royal Children's Hospital Melbourne; Jurgen de Graaff, Erasmus Medical Centre; Nicola Disma, Gaslini Children's Hospital; Mary Ellen McCann and Charles Berde, Children's Hospital Boston; Francis McGowan and Dr Christopher Ward, The Children's Hospital of Philadelphia; Justin Skowno, The Children's Hospital at Westmead; Beverley Orser, University of Toronto and SmartTots representative; Daniel Sessler, Cleveland Clinic; Peter Szmuk, Children's Medical Center Dallas; Paul Lee Archer, Queensland Children's Hospital; Britta von Ungern Sternberg, Perth Children's Hospital; Laszlo Vutskits, University Hospital of Geneva.) granted approval for analysis of pharmacokinetic data from the Italian Investigators.

## FUNDING INFORMATION

Funding was provided by Italian Agency for Medications (AIFA) to cover the cost of patients enrolled in Italy (AIFA‐TRS‐2018‐00001250).

## CONFLICT OF INTEREST

Brian Anderson is Associate Editor‐in‐Chief for Pediatric Anesthesia. Nicola Disma is Associate Editor for Pediatric Anesthesia.

## Clinical trial number and registry URL

ClinicalTrials.gov Identifier: NCT03089905. EudraCT number: 2017‐002803‐81.

## ETHICS STATEMENT

Comitato Etico Regionale Liguria n. 365/2019.

## Supporting information


Appendix S1

## Data Availability

The data that support the findings of this study are available from the corresponding author upon reasonable request.
