# Supplementary material for: Justification of empiric methodology to determine dexmedetomidine dose for the TREX study
Source: Paediatr Anaesth. 2022 Dec 4;33(3):236–42. doi: 10.1111/pan.14605 (PMC10107467; doi:10.1111/pan.14605)
Supplement: Supplementary file 1 — Appendix S1 [file PAN-33-236-s001.docx]

# **Supplementary material**

**Dexmedetomidine PK model TBW**

$PROB dexmedetomidine universal

$INPUT ID TIME AMT DUR RATE MDV DVID DV AGEY PMAW WTKG FFMKG HTCM BMI M1F0 GRP STUDY

$DATA Dexmed_Potts_Cortinez_Talke_Hannivoort_18.csv IGNORE #

IGNORE(GRP.EQ.3) ;IGNORE FDIAZ

$ESTIM MAXEVAL=9999 NSIG=3 SIGL=9 PRINT=1 NOABORT METHOD=CONDITIONAL INTERACTION

MSFO=dexmede_uni.msf

$THETA 1. FIX ;POP_FFATCL

$THETA 1. FIX ;POP_FFATV

$THETA (0.001,0.641, 15) ; POP_CL

$THETA (0.5,20.7,500) ; POP_V1

$THETA (0.008,1.22,50) ; POP_Q2

$THETA (5,37.4,500) ; POP_V2

$THETA (0.001,0.28,10) ; POP_Q3

$THETA (5,39.1,150) ; POP_V3

$THETA (0,45.7,500) ; POP_TM50CL

$THETA (0.5,1.81,5) ; POP_HILLCL

;RESIDUAL UNIDETIFIED VARIABILITY ON PLASMA DEX CONCENTRATIONS

$THETA (0.001,0.202,) ;RUV_CVCP

$THETA (0.001,0.00408, ) ;RUV_SDCP

$OMEGA BLOCK(6)

0.104 ; PPVCL

0.0559 0.884 ; PPVV1

0.0353 -0.415 0.314 ; PPVQ2

0.046 -0.147 0.204 0.255 ; PPVV2

0.222 -0.0743 0.232 0.0656 0.966 ; PPVQ3

0.169 -0.51 0.485 0.383 0.578 1.02 ; PPVV3

$OMEGA 0.1 ; PPV_RUVCP

$SIGMA 1. FIX ;EPS1

$SUBROUTINE ADVAN13 TOL=9

$MODEL

COMP(CENTRAL)

COMP(PERIPH1)

COMP(PERIPH2)

$PK

LN2=LOG(2)

FFATCL=POP_FFATCL;*EXP(PPV_FFATCL)

FFATV=POP_FFATV;*EXP(PPV_FFATV)

NFMCL=FFMKG + FFATCL*(WTKG - FFMKG) ; NFM FOR CLEARANCE

NFMV=FFMKG + FFATV *(WTKG - FFMKG) ; NFM FOR VOLUME

FSZCL=(NFMCL/70)**(3/4)

FSZV=(NFMV/70)

TMHCL=POP_TM50CL**POP_HILLCL

PMAHIL=PMAW**POP_HILLCL

CLAGE=PMAHIL/(PMAHIL+TMHCL)

CL=FSZCL*CLAGE*POP_CL*EXP(PPVCL)

Q2=FSZCL*POP_Q2*EXP(PPVQ2)

Q3=FSZCL*POP_Q3*EXP(PPVQ3)

V1=FSZV*POP_V1*EXP(PPVV1)

V2=FSZV*POP_V2*EXP(PPVV2)

V3=FSZV*POP_V3*EXP(PPVV3)

S1=V1

S2=V2

S3=V3

$DES

DCP=A(1)/V1

DC2=A(2)/V2

DC3=A(3)/V3

DADT(1)=DC2*Q2+DC3*Q3-DCP*(CL+Q2+Q3)

DADT(2)=Q2*(DCP-DC2)

DADT(3)=Q3*(DCP-DC3)

$ERROR

CP=A(1)/V1 ;DEXMEDETOMIDINE PLASMA CONCENTRATION

IF(DVID.EQ.1)THEN ;DEXMEDETOMIDINE PLASMA CONCENTRATIONS

PROPCP=CP*RUV_CVCP

ADDCP=RUV_SDCP

SDCP=SQRT(PROPCP*PROPCP + ADDCP*ADDCP)*EXP(PPV_RUVCP)

Y=CP + SDCP*EPS1

ENDIF

$TABLE ID TIME WTKG CL V1 Q2 V2 Q3 V3 Y DVID AGEY MDV

ONEHEADER NOPRINT FILE=dexmede_all.fit
